# Interstitial lung disease in patients with systemic lupus erythematosus: a cohort study

**DOI:** 10.3906/sag-2109-16

**Published:** 2021-12-21

**Authors:** Naci ŞENKAL, Esen KIYAN, Ali Aslan DEMİR, Yasemin YALÇINKAYA, Ahmet GÜL, Murat İNANÇ, Mahmude Lale ÖÇAL, Bahar ARTIM ESEN

**Affiliations:** 1 Department of Internal Medicine, İstanbul Faculty of Medicine, İstanbul University, İstanbul, Turkey; 2 Department of Pulmonary Medicine, İstanbul Faculty of Medicine, İstanbul University, İstanbul, Turkey; 3 Fulya Radiologic Imaging Center, İstanbul, Turkey; 4 Department of Internal Medicine, Division of Rheumatology, İstanbul Faculty of Medicine, İstanbul University, İstanbul, Turkey

**Keywords:** SLE, interstitial lung disease, fibrosis, spirometry, carbon monoxide diffusion test

## Abstract

**Background/aim:**

Systemic lupus erythematosus (SLE) is an autoimmune disease with a variety of organ/system involvement. Respiratory system involvement is common in these patients and usually manifests itself by disorders of the lung parenchyma, pleura, pulmonary vasculature or diaphragm. In this study, we sought to determine the frequency of interstitial lung disease (ILD) in patients with SLE and associated risk factors.

**Materials and methods:**

Three hundred randomly chosen patients with SLE were included. Chest x-ray (CXR), lung spirometry and carbon monoxide diffusion test (DLCO) were performed. High-resolution thorax computed tomography (HRCT) was performed for a definite diagnosis of ILD.

**Results:**

Of 300 patients, 16% had ILD. At the start of the study, the prevalence obtained from the patients’ records showed that 4% had ILD. The median age, mean duration of disease, and follow-up time were significantly higher and longer in patients with ILD compared to patients without (p < 0.05). Forced expiratory volume (FEV1), forced vital capacity (FVC), DLCO and total lung capacity (TLC) were significantly lower in patients with ILD (p < 0.001). Patients with ILD had a significantly higher frequency of arthritis, serositis, Raynaud’s phenomenon, myositis, and anti-Scl70 positivity (p = 0.01, 0.001, 0.02, 0.004, and 0.001, respectively). A significantly higher number of patients had stopped using hydroxychloroquine (HCQ) in the ILD group (p = 0.04).

**Conclusion:**

ILD is common in patients with SLE. Spirometry, diffusion tests, and CXR are simple but valuable tools to diagnose ILD in patients with SLE. Considering the significant difference of prevalence between the start and the end of the study, one of the possibilities is the underrecognition of SLE-associated interstitial pulmonary disease. The higher administration of immunosuppressives in these patients may support a multisystemic active disease including the lungs.

## 1. Introduction

Systemic lupus erythematosus (SLE) is a multisystemic chronic autoimmune disease characterized by remissions and exacerbations [1]. Pulmonary involvement develops in up to 50% of patients during the course of SLE. As the clinical presentation is often insignificant and nonspecific, pulmonary involvement may be overlooked.

Clinically significant interstitial lung disease (ILD) occurs in 1%–15% of patients with SLE. These patients may present with an asymptomatic course or typically present with exertional dyspnea and nonproductive cough. The asymptomatic course demonstrates the importance of pulmonary function tests and thoracic imaging. In previous studies, long disease duration, late onset disease, Raynaud’s phenomenon, anti-(U1) RNP antibody positivity, sclerodactyly, and abnormal nail capillary structure were determined as risk factors for the development of ILD in SLE. High CRP, cryoglobulins, hypocomplementemia and antinuclear antibody (ANA) positivity were found to be associated with ILD in these patients [2–5].

The diagnosis is based on a combination of clinical features, imaging, histopathology and lung physiology. Although lung biopsy is the gold standard method for diagnosing ILD, it is usually not performed due to its invasive nature. Nonspecific interstitial pneumonia (NSIP) is the most common histological and radiologic pattern of SLE-ILD whilst organizing pneumonia (OP), lymphoid interstitial pneumonia (LIP), ordinary interstitial pneumonia (UIP), desquamative interstitial pneumonia (DIP), and diffuse alveolar damage may also be seen. High-resolution lung tomography (HRCT), pulmonary function test (PFT), and carbon monoxide diffusion test (DLCO) can aid to diagnose ILD instead of invasive diagnostic methods [3,4].

Herein, we aimed to assess the frequency of ILD in patients with SLE followed up at a dedicated lupus clinic and determine the associated risk factors.

## 2. Materials and methods

### 2.1. Participants

This study is conducted between January 2019 and January 2020. Three hundred patients were consequently chosen from those followed up at the SLE/Antiphospholipid syndrome (APS) outpatient clinic during their routine follow-up dates.

All fulfilled the SLICC and ACR SLE classification criteria [6]. Patient demographics, clinical and laboratory characteristics, data regarding disease damage (SLICC/SDI) and treatment histories were obtained from the Rheumatology Department SLE/APS patient registry and were revised. A special focus was given on the frequency of interstitial lung disease defined by the presence of this data on the database and the patient follow-up notes at the start of the study. Subsequently, all patients were assigned to undergo spirometric measurement and carbon monoxide diffusion test.

Informed consent was obtained from all patients. The study was approved by the İstanbul Faculty of Medicine Ethics Committee with the number 2018/1758 and was conducted in accordance with the standards specified in the World Medical Association Helsinki Declaration.

### 2.2. Spirometric evaluation and carbon-monoxide diffusion test

Spirometric measurements (FVC, FEV1, FEV1/FVC), total lung capacity (TLC) measurements and diffusing capacity of the lungs for carbon monoxide (DLCO) measurements were performed in all subjects participating in the study in accordance with the recommendations of the American Thoracic Society and the European Respiratory Society [7–9]. The “single breath method” was used for TLC and DLCO measurements. DLCO measurements were corrected for serum hemoglobin (DLCO) [10]. FVC, FEV1, FEV1/FVC, DLCO, and TLC values that were higher than 80% of the healthy average were considered normal [11].

### 2.3. Diagnosis of interstitial lung diseases

Chest X-rays (CXR) performed within the last 6 months were evaluated. If no CXR was available in the defined period of time, the patient underwent CXR. If an undiagnosed patient had an unexplained low FVC or DLCO and/or suspected findings on CXR, a high resolution computed tomography (HRCT) was performed. Images were evaluated independently by a radiologist and a pulmonologist who were blinded to the clinical parameters. After excluding other possible causes, reticular and/or interstitial pattern, ground-glass opacity, patchy consolidation, or honeycomb appearance were assessed as ILD.

### 2.4. Statistical analysis

Normal distribution was assessed using Kolmogorov-Smirnov test. Differences of continuous variables between groups were evaluated using t test or Mann-Whitney U test. Categorical variables were compared using chi-square tests. Statistical significance was accepted as p < 0.05. Continuous variables were expressed as mean ± standard deviation (SD) and categorical variables were expressed as percentages (%). Analyses were performed using the SPSS (SPSS version 22.0, IBM Corp., Armonk, New York, USA) software.

## 3. Results

### 3.1. Demographic and clinical features

Three hundred patients with SLE followed up in the SLE/APS outpatient clinic were evaluated. Eighty-eight percent were female. The median age of the patients was 42 (19–76) and the median age at the time of diagnosis was 30 (7–69). The median duration of the disease and follow-up were 11 (1–39) and 10 (1–39) years, respectively. The demographic and detailed clinical characteristics of the patients are shown in table 1. According to the data obtained from the database and patient follow-up notes before investigations regarding ILD were started, the frequency of ILD in this cohort was %4 (table 1).

Regarding treatment, 17% (n = 51) of the cohort had stopped taking HCQ due to retinal toxicity. The mean duration of HCQ use was 9.8 ± 6.8 (1–30) years. Fifty-five percent of the patients (n = 165) had received azathioprine treatment (mean duration of use 6.8 ± 4.8 [1–20] years); 46% (n = 137) mycophenolate mofetil (MMF) treatment (mean duration 4.9 ± 3.4 [1–15] years); 43.3% (n = 130) cyclophosphamide (mean cumulative dose 4.8 ± 2.8 [1–15] g); 4.3% had received (n = 13) methotrexate and 17% (n = 51) had received rituximab (mean number of cycles 2.6 ± 1.7 [1–8]).

### 3.2. Spirometric measurement and carbon monoxide diffusion test

The mean FVC value of the whole cohort was found as 96.1% (37–147), FEV1 as 92.9% (39–145), FEV1/FVC as 82.2% (60–105), DLCO as 80.9% (37–123), and TLC as 94.8% (50–127).

According to the results of chest X-ray (CXR) and spirometric measurement/DLCO tests, 48 (16%) patients were considered to have ILD. The mean FVC of patients with ILD was 86% (50–126), FEV1 was 83.4% (47–125), DLCO was 63.6% (37–89) and TLC was 82.9% (50–14.1). Compared to patients without ILD, these values were significantly lower (p < 0.001 for all comparisons) (Table 2).

### 3.3. Patients with ILD

In patients a showing restrictive pattern or/with lower levels of DLCO, diagnosis of ILD was made by the detection of interstitial appearance in posterior anterior (PA) CXR or by thorax HRCT in the case of a suspicious appearance in the PA CXR. Forty-eight patients were identified to definitely have ILD with a dominance of nonspecific interstitial pneumonia pattern followed by usual interstitial pneumonia pattern in a very small fraction of patients.

The comparison of SLE patients with (n = 48) and without (n = 252) ILD in terms of demographic characteristics are shown in Table 3. The median age, mean disease, and follow-up time of SLE-ILD (+) patients were significantly higher and longer than those of patients without ILD (p = 0.003, 0.01, and 0.02, respectively).

The frequency of arthritis and serositis was significantly higher in the SLE-ILD (+) group (89.6% vs 73, p = 0.01; 41.7% vs 24.6, p = 0.001 respectively). The frequency of Raynaud’s phenomenon and the presence of myositis were also significantly higher in the SLE-ILD (+) group (52.1% vs 34.9, p = 0,02); 14.6% vs 3.2, p = 0.004, respectively). Anti-Scl70 antibodies positivity rate was higher in the group with SLE-ILD (+) (12.5% vs 1.2, p = 0.001).

The mean damage score of SLE-ILD (+) patients was 2.8 ± 1.7, and 0.9 ± 1.3 in patients without ILD (p < 0.001). Neuropsychiatric damage (31.3% vs 8.7, p < 0.001), avascular necrosis (22.9% vs 9.9, p = 0.02) and diabetes (16.7 vs 3.6, p = 0.002) were significantly higher in SLE-ILD (+) patients. There was no significant difference between the two groups in terms of renal and cardiovascular damage.

During the follow-up period of SLE-ILD (+) patients, 4 patients (8 %) developed malignancy whilst 5 in (2%) SLE-ILD (–) were diagnosed with cancer (p = 0.04). Regarding the types of malignancy, 1 had thyroid papillary cancer, 1 had breast cancer, 1 had severe dysplasia of the vocal cord and 1 had diffuse large B-cell lymphoma. The frequency of smokers in patients with ILD was significantly higher compared to patients without (35.4% vs 15.8%, p = 0.002). Comparison of treatment in both groups revealed that the discontinuation rate of HCQ was higher in SLE-ILD (+) group (27.1% vs 15.1, p = 0.04). There were no differences in the frequencies of azathioprine, cyclophosphamide, rituximab, and methotrexate use. However, the use of MMF was significantly higher in the ILD (+) group (62.5% vs 42.9, p = 0.01) (Table 4).

## 4. Discussion

SLE is a chronic autoimmune disease with multisystemic involvement, characterized by remissions and exacerbations [1]. The frequency of lung disease in SLE varies in different series with up to 50% of involvement [3]. Pulmonary involvement may not always show up with remarkable clinical symptoms, but can be silent or present with a progressively symptomatic pattern as may be the case with ILD [3]. Moreover, diagnosis may be shadowed in the setting of a multisystemic active disease with a noisy presentation leading to a shift in physician’s focus. In this study, we aimed to determine the frequency and characteristics of patients with ILD in a cohort of patients with SLE and reveal the factors associated with it.

The frequency of ILD at the beginning of the study as obtained from the database and the patient records and frequency at the end of our study was remarkably different, with a higher percentage in the latter (4% vs 16%). Although the existence of a multisystemic active disease leading the clinician to start a prompt treatment focusing on vital organs with relatively easily noticed involvement signs and the lack of sufficient investigations to diagnose ILD may be explanations for this significant difference, it is also important to consider the possible contribution of drug effect to the occurrence of ILD.

Narvaez et al. in the Spanish multicenter RELESSER cohort [12] published in 2018, reported that pleuropulmonary involvement was associated with long disease duration, advanced patient age, presence of Raynaud’s phenomenon, and neuropsychiatric involvement, as found similarly in our study. The frequency of ILD in this cohort was 2.4% and the mean duration of the disease up to the diagnosis of ILD was 7.7 years. These patients were reported to have significantly lower survival. Pulmonary involvement was found to be associated with the presence of antiphospholipid antibodies, severe nephritis, nonischemic heart disease, vasculitis, hematological involvement, and anti-RNP antibodies. Importantly a high SLEDAI score was positively correlated with pleuropulmonary involvement in this cohort.

Haye Salinas et al. in the GLADEL cohort [13] published in 2017 reported at least one pleuropulmonary involvement in 421 patients (28.4%) of 1480 retrospective SLE patients. The frequency of ILD was 1.4%, lower than the rates reported in clinical studies and autopsy series and an association was found with longer disease duration.

In a study published in 2002 by Nakano et al [14], spirometry, PA CXR, and HRCT results of 110 patients were retrospectively examined. As a result, DLCO was found to be decreased in 52 patients (47%), and a restrictive pattern was found in 9 patients (8%) as a result of spirometric tests. The prevalence of pulmonary fibrosis was found to be 13% and was correlated with the presence of Raynaud’s phenomenon, similar to our study.

Significantly increased prevalence of Raynaud’s phenomenon in ILD may be a warning sign for the presence of ILD. The possibility of an overlap with systemic sclerosis should also be considered in these patients. Simple pulmonary function tests may give a clue for the existence of ILD. However, as false negative test results may also be the case, capillaroscopy and thoracic HRCT in suspicious cases may be considered for an early diagnosis.

In the study published by Enomoto et al. in 2019 [15], 55 patients with a diagnosis of SLE-ILD were retrospectively examined and smoking was found to be significant. Other parameters associated with SLE-ILD were thrombocytopenia and high anti-ds DNA titers [15]. We also found that a significant proportion of patients with ILD were smokers.

In our cohort, a small number of patients with ILD were found to have myositis and/or anti-Scl-70 antibody positivity. These patients all met the SLICC and ACR SLE classification criteria, myositis specific antibodies were not tested and none could be classified as systemic sclerosis initially as none were known to have ILD before the study. This may show the importance of a low threshold to investigate ILD in patients with myositis and/or anti-Scl70 positivity even if there are no symptoms of pulmonary disease.

Among patients with damage, the mean damage score in patients with ILD was significantly higher compared to patients without in our cohort. Regarding the comparison of damaged items, the frequency of damage attributable to glucocorticoids such as diabetes and avascular necrosis was significantly higher in the ILD+ group. Despite the lack of data on disease activity and cumulative steroid dose, this finding may be interpreted as indirect support for higher disease activity in patients with ILD requiring a higher dose of glucocorticoids.

MMF use was significantly higher in our patients with ILD. Since there is no data regarding the presence of ILD at the initiation of MMF treatment, it is not possible to establish a causal relationship between the drug and ILD occurrence. On the other hand, ILD might be a part of multisystemic involvement requiring MMF treatment, the use of which might have prevented/delayed the emergence of ILD symptoms and signs.

HCQ is an important immunomodulatory drug licensed in the treatment of SLE and controls disease activity. It has been shown to reduce damage and mortality in patients with SLE [16, 17]. We found that a higher number of patients discontinued HCQ treatment due primarily to retinal toxicity in the ILD+ group. Although we do not know the exact time of onset for ILD, it is possible to interpret this finding as the capability of HCQ to prevent the development of ILD as the drug has many beneficial effects. The threshold for drug discontinuation should be kept high, and its continuation should be encouraged unless toxicity is certainly proved.

The most important limitation of our study is the lack of an inception cohort where it could be possible to detect the first occurrence of ILD. Since a significant portion of the patients were inactive or had low disease activity at the time of the study, it is difficult to determine a relationship with the disease activity and it is not possible to make a definite judgment since the disease activity at the onset of lung involvement is not known. Also important is to consider the possible contribution of drug effect to the occurrence of ILD. Since data before the onset of treatment is lacking in most cases, this possible effect cannot be shown.

## 5. Conclusion

In conclusion, considering the remarkable difference of the frequency of ILD at the beginning and the end of the study with a higher in the latter, we think that the detection of the real incidence and prevalence of ILD will ideally be possible by performing periodic screenings in the follow up of patients with SLE starting from baseline. In clinical practice, special consideration should be given to diagnose ILD as it can be overlooked especially in the case of a multisystemic active disease. Simple spirometry-DLCO tests and PA CXR during the routine controls of the patients will help to decide on the necessity of further investigations.

## Figures and Tables

**Table 1:** Clinical, laboratory, and demographic characteristics of SLE patients.

SLE n = 300	
Female, n (%)	265 (88.3)
Age (years) (mean ± SD, IQR)	43 ± 12.5 (19–76)
Age at diagnosis (years) (mean ± SD, IQR)	32 ± 12.2 (7–69)
Disease duration (years) (mean ± SD, IQR)	12.5 ± 8.3 (1–39)
Follow-up period (years) (mean ± SD, IQR)	11.5 ± 8.03 (1–39)
Clinical Features, n (%)	
Photosensitivity	196 (65.3)
Alopecia	139 (46.3)
Malar rash	162 (54)
Discoid rash	23 (7.7)
Oral ulcer	32 (10.7)
Arthritis	227 (75.7)
Serositis (total)	82 (27.3)
Pericarditis	53 (17.7)
Pleuritis	54 (18)
Renal involvement	128 (42.7)
Hematological involvement (total)	219 (73)
Leukopenia	120 (40)
Lymphopenia	185 (61.7)
Thrombocytopenia	86 (28.7)
AIHA	78 (26)
Neurological involvement (total)	21 (7)
Seizure	13 (4.3)
Psychosis	8 (2.7)
Anti-dsDNA	242 (80.7)
ANA positivity	299 (99.7)
Anti-Sm	79 (26.3)
ACA IgG	50 (16.7)
ACA IgM	49 (16.3)
Lupus anticoagulant	68 (22.7)
Raynaud phenomenon	113 (37.7)
APS	65 (21.7)
Arterial thrombosis	43 (14.3)
Vein thrombosis	29 (9.7)
Livedo reticularis	53 (17.7)
Myositis	15 (5)
Interstitial lung disease*	12 (4)
Ischemic heart disease	6 (2)
Vasculitis	43 (14.3)
Anti-RNP	64 (21.3)
Anti-Ro	94 (31.3)
Anti-La	26 (8.7)
Scl-70	9 (3)
RF	20 (6.7)
Anti-CCP	8 (2.7)

ACA: Anticardiolipin antibody, AIHA: Autoimmune hemolytic anemia, ANA: Antinuclear antibody, Anti-CCP: Anti cyclic citrullinated peptide, Anti-RNP: Antiribonucleoprotein antibodies, APS: Antiphospholipid syndrome, Anti-Sm: Anti-Smith, RF: Rheumatoid factor, SLE: Systemic lupus erythematosus.

* According to the data obtained from the database and patient follow-up notes before investigations regarding ILD were started.

**Table 2:** Spirometry-DLCO characteristics of the SLE-ILD patient group.

Test	SLE-ILD (−) (n=252)	SLE-ILD (+) (n=48)	P value^1^
FVC% mean ± SD (min-max)	98 ± 15.3 (37–147)	86 ± 17.1 (50–126)	<0.001^*^
FEV1% mean ± SD (min-max)	94.7 ± 15.3 (39–145)	83.4 ± 17 (47–125)	<0.001^*^
FEV1/FVC mean ± SD (min-max)	82.3 ± 6.1 (60–105)	81.7 ± 4.7 (72–94)	0.4
DLCO% mean ± SD (min-max)	84.2 ± 12.2 (45–123)	63.6 ± 11.8 (37–89)	<0.001^*^
TLC% mean ± SD (min-max)	97.1 ± 12.7 (50–127)	82.9 ± 14.1 (54–108)	<0.001^*^

DLCO: Carbon monoxide diffusion capacity, FEV1: Forced expiratory volume at 1st s, FVC: Forced vital capacity, ILD: Interstitial lung disease, max: Maximum, min: Minimum, mean: Average, SD: Standard deviation, SLE: Systemic lupus erythematosus, TLC: Total Lung Capacity

1 Mann-Whitney U test

**Table 3:** Demographic characteristics of SLE-ILD (+) and ILD (–) groups.

Features	SLE-ILD (−) (n = 252)	SLE-ILD (+) (n = 48)	P value^1^
Gender, female n (%)	222 (88.1)	43 (89.6)	0.7
Age mean ± SD (min-max)	42 ± 12.2 (19–76)	48 ± 12.6 (25–71)	0.003^*^
Age at diagnosis mean ± SD (min-max)	31 ± 12 (7–69)	34 ± 13 (15–65)	0.19
Disease duration, years, mean ± SD (min-max)	11.9 ± 8 (1–39)	15.3 ± 9.3 (1–35)	0.01^*^
Follow-up period, year, mean ± SD (min-max)	11 ± 7.7 (1–39)	14.1 ± 8.8 (1–34)	0.02^*^

ILD: Interstitial lung disease, max: Maximum, min: Minimum, mean: Average, SD: Standard deviation, SLE: Systemic lupus erythematosus.

1 Mann-Whitney U test.

**Table 4:** Drug use in patients with and without SLE-ILD.

Features, n (%)	SLE-ILD (−) (n = 252)	SLE-ILD (+) (n = 48)	P value^1^
AZA	138 (54.8)	27 (56.3)	0.8
MMF	108 (42.9)	30 (62.5)	0.01^*^
CYC	103 (40.9)	27 (56.3)	0.05
HCQ discontinuation	38 (15.1)	13 (27.1)	0.04^*^
Rituximab	39 (15.5)	12 (25)	0.1
Methotrexate	11 (4.4)	2 (4.2)	1

AZA: Azathioprine, CYC: Cyclophosphamide, HCQ: Hydroxychloroquine, ILD: Interstitial lung disease, MMF: Mycophenolate mofetil, SLE: Systemic lupus erythematosus.

1 Pearson’s chi-square test.
